# Association of Pathway Mutations With Survival in Taiwanese Breast Cancers

**DOI:** 10.3389/fonc.2022.819555

**Published:** 2022-07-22

**Authors:** Po-Sheng Yang, Ying-Ting Chao, Chun-Fan Lung, Chien-Liang Liu, Yuan-Ching Chang, Ker-Chau Li, Yi-Chiung Hsu

**Affiliations:** ^1^ Department of Medicine, MacKay Medical College, New Taipei City, Taiwan; ^2^ Department of General Surgery, MacKay Memorial Hospital, Taipei, Taiwan; ^3^ Department of Biomedical Sciences and Engineering, National Central University, Taoyuan, Taiwan; ^4^ Institute of Statistical Science, Academia Sinica, Taipei, Taiwan; ^5^ Department of Statistics, University of California Los Angeles, Los Angeles, CA, United States

**Keywords:** cancer panel, next-generation sequencing, breast cancer, survival analysis, triple negative

## Abstract

Breast cancer is the most common invasive cancer in women worldwide. Next-generation sequencing (NGS) provides a high-resolution profile of cancer genome. Our study ultimately gives the insight for genetic screening to identify the minority of patients with breast cancer with a poor prognosis, who might benefit from the most intensive possible treatment. The detection of mutations can polish the traditional method to detect high-risk patients who experience poor prognosis, recurrence and death early. In total, 147 breast cancer tumors were sequenced with targeted sequencing using a RainDance Cancer Hotspot Panel. The average age of all 147 breast cancer patients in the study was 51.7 years, with a range of 21–77 years. The average sequencing depth was 5,222x (range 2,900x-8,633x), and the coverage was approximately 100%. A total of 235 variants in 43 genes were detected in 147 patients by high-depth Illumina sequencing. A total of 219 single nucleotide variations were found in 42 genes from 147 patients, and 16 indel mutations were found in 13 genes from 84 patients. After filtering with the 1000 Genomes database and for synonymous SNPs, we focused on 54 somatic functional point mutations. The functional point mutations contained 54 missense mutations in 22 genes. Additionally, mutation of genes within the RET, PTEN, CDH1, MAP2K4, NF1, ERBB2, RUNX1, PIK3CA, FGFR3, KIT, KDR, APC, SMO, NOTCH1, and FBXW7 in breast cancer patients were with poor prognosis. Moreover, TP53 and APC mutations were enriched in triple-negative breast cancer. APC mutations were associated with a poor prognosis in human breast cancer (log-rank P<0.001). Our study identified tumor mutation hotspot profiles in Taiwanese breast cancer patients, revealing new targetable gene mutations in Asian breast cancer patients.

## Introduction

Breast cancer is the most common form and leading cause of cancer death for women worldwide, with nearly 45,000 deaths each year (1, 2), and it is also the highest incidence of all cancers in women in Taiwan (3). In the US, breast cancer is the most common cancer among women, and women have a 12% lifetime risk of developing breast cancer (4). Breast carcinoma is known to arise from a multifactorial process and is approximately twice as common in first-degree relatives of women with the disease as in the general population, consistent with genetic susceptibility to the disease. More frustrating from a cancer prevention viewpoint is that 40% of Taiwanese women who develop this disease fit none of the currently identified risk groups. This has prompted us to search for clues at the molecular level that may help in understanding breast tumor pathogenesis. Breast cancer is a heterogeneous disease, appearing in a variety of forms with different behaviors (5–7). Next-generation sequencing technology is based on massively parallel sequencing of millions of fragments using novel reversible terminator-based sequencing chemistry. This new technology provides high-throughput measurements of the whole genome sequence, single nucleotide polymorphisms (SNPs), copy number variations, insertions, and deletions and can be performed *de novo*, on the transcriptome or on targeted sequences.

In past few years, many genomes have been sequenced, and this has rapidly generated large volumes of genomic data (8, 9). Several large-scale genomic studies have revealed the heterogeneity of breast cancer (10–12), and these experiments provide rich genetic information for breast cancer research. Different targeted next-generation sequencing (NGS) panels have been used in breast cancer studies (13–15). The gene mutations have been different in different reported studies. Therefore, the genetic profiles might differ between geographic or ethnic populations (16). However, the genetic spectrum of Asian breast cancer remains limited, and the most common breast cancer is not well studied in the Taiwanese population. We used the RainDance Cancer Hotspot Panel for 147 Taiwanese breast cancer patients. The cancer panel contains 54 genes, including numerous known key driver genes (BRCA1, BRCA2 and ERBB2) in breast cancer. In our study, we focused on the relationship between somatic mutations and survival. The results will be useful for clinical prognosis and therapeutic target development. Cancer is a complex genetic disease driven by somatic mutations that accumulate in the genome during the lifetime of an individual. These somatic mutations could also additionally contribute to transcriptomic alterations, single nucleotide variants (SNVs), insertions and deletions (indels), copy number variations, and genome rearrangements (structural variants, SVs) (17). We hope our study ultimately paves the way for genetic screening to identify the minority of breast cancer patients with a poor prognosis, who might benefit from the most intensive possible treatment. The detection of mutations can improve our ability to detect high-risk patients who are likely to experience relapse and die early.

## Materials and Methods

### Clinical Patients

A total of 147 pathologically confirmed breast cancer samples were obtained from the tissue bank of MacKay Memorial Hospital. This investigation was performed after approval by the Institutional Review Board (MacKay Memorial Hospital: 12MMHIS121 and Academia Sinica: AS-IRB02-102086). The clinical information is provided in **Supplementary Table 1**.

### Targeted Enrichment of Genomic DNA Regions for Next-Generation Sequencing

To identify rare and novel variants in genes associated with cancer, we used a microdroplet-based target enrichment (RainDance Technologies, Billerica, MA) hotspot cancer panel followed by high-throughput sequencing. A primer library for 54 susceptible genes related to cancer was designed using a proprietary pipeline. The primer library was combined dropwise with each sheared genomic DNA sample on a RainDance ThunderStorm™ Panel (RainDance). Microdroplet-based PCR of 54 genes targeting more than 13,000 known mutations found in the Catalogue of Somatic Mutations in Cancer (COSMIC) database was performed. All 40 samples enriched with targeted amplicons were sequenced on the MiSeq platform (Illumina, Inc., San Diego, CA), generating 250-bp paired-end reads according, to the manufacturer’s instructions. NGS raw sequencing data were submitted to the NCBI Sequence Read Archive with BioProject ID PRJNA758602.

### Variant Calling

Reads were aligned against the human reference genome (UCSC Hg19) using BWA (7.5.a) (18). Variants called by GATK software (2.7-2) (19) and SAMtools software (0.1.19) (20) were annotated using the Annovar package (21).

### Functional Enrchment Analysis of Genes With Somatic Mutations

We used Ingenuity Pathway Analysis (IPA) (QIAGEN company, CA), a web-based computational platform, to conduct functional enrichment analysis of genes. We input the set of genes with somatic mutations and used the core analysis enrichment tool with the default settings.

### Sanger Sequencing Validation of APC Mutations

For APC mutations prioritized for Sanger validation, we designed PCR primers and validated the presence of candidate mutations by Sanger sequencing on samples using a 3500xL DX Genetic Analyzer (Life Technologies Co., Grand Island, New York).

### Survival Analysis

We calculated the patients’ risk scores from the number of functional mutations and classified them into high-risk or low-risk groups with the medium risk score as the threshold. Kaplan-Meier survival curves were obtained and compared by log-rank tests.

### Statistical Analysis

Samples with mutations and without mutations for each clinical phenotype were determined using 2 × 2 contingency tables, and Fisher’s exact test was used to calculate P-values in the detected mutated genes. All P-values were two sided, and statistical significance was defined as P < 0.05.

## Results

### The Cancer Hotspot Mutation Profile of Breast Cancer

**Figure 1:**
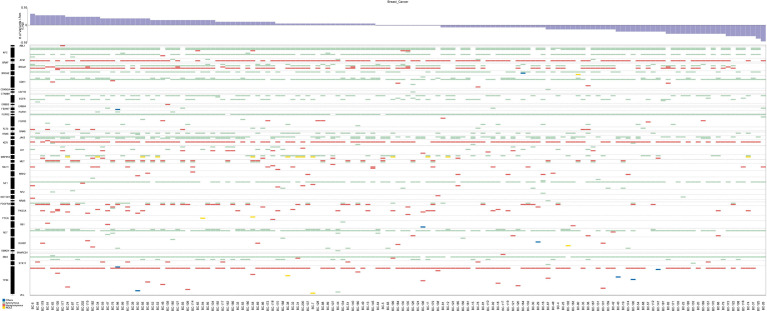
Mutations and in the 147 breast cancer patients. The top panel shows a summary of the mutations in 43 cancer hotspot genes (see text and **Table S2** for details). Patients are arranged from left to right by the number of mutations, shown in the top track. Colored rectangles indicate the mutation category observed in a given gene.

In total, 147 breast cancer patients underwent targeted sequencing with the RainDance Cancer Hotspot Panel. The average age of all 147 breast cancer patients included in the study was 51.7 years, with a range of 21–77 years. Breast cancer samples were categorized as ER-positive, HER2-positive, PR-positive and triple-negative subtypes (**Table 1**). The average sequencing depth was 5,222x (range 2,900x-8,633x), and the coverage was approximately 100% (**Table S2**). A total of 235 variants in 43 genes were detected in 147 patients by high-depth Illumina sequencing (**Figure 1**). A total of 219 single nucleotide variations were found in 42 genes from 147 patients, and 16 indel mutations were found in 13 genes from 84 patients. After filtering with the 1000 Genomes database and for synonymous SNPs, we focused on 54 somatic functional point mutations. The functional point mutations contained 54 missense mutations in 22 genes.

**Table 1:** Clinical characteristics of the 147 breast cancer patients.

Variable	Characteristic	n (%)
Age	≦50 years	77 (52.4%)
	>50 years	70 (47.6%)
Stage	0	3 (2.0%)
	1	46 (31.3%)
	2	70 (47.6%)
	3	28 (19%)
ER	Positive	97 (66.0%)
	Negative	50 (34.0%)
PR	Positive	75 (51.0%)
	Negative	72 (49.0%)
Her2	Negative	105 (71.4%)
	Positive	42 (28.6%)
ER, PR & HER2 Status	Triple negative	29 (19.7%)
	Non-TN	118 (80.3%)

### The Missense Somatic Variant Allele Frequency

**Figure 2:**
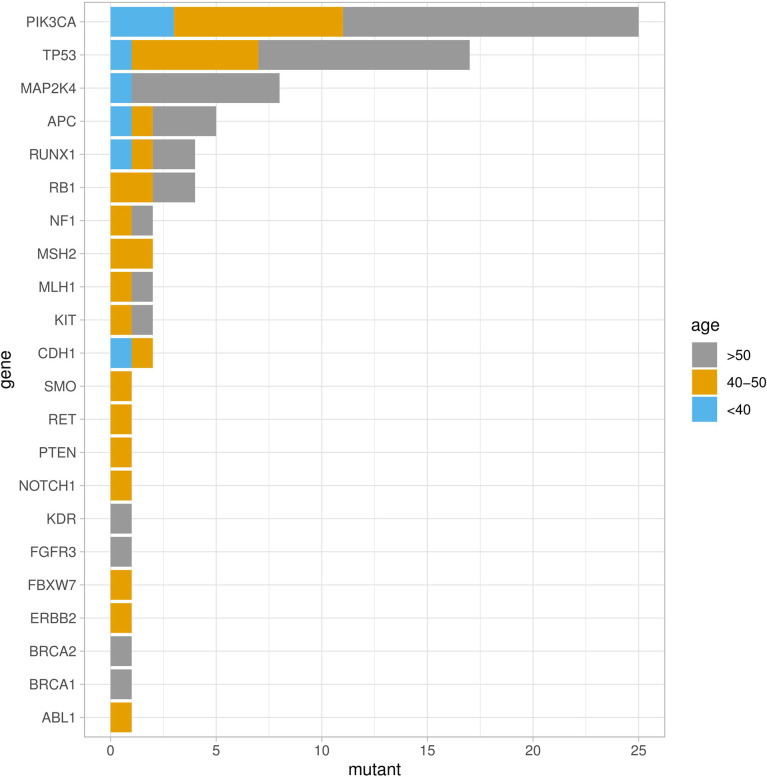
Somatic missense mutation profiles of breast cancers identified by a hotspot panel of 22 cancer-associated genes.

**Figure 3:**
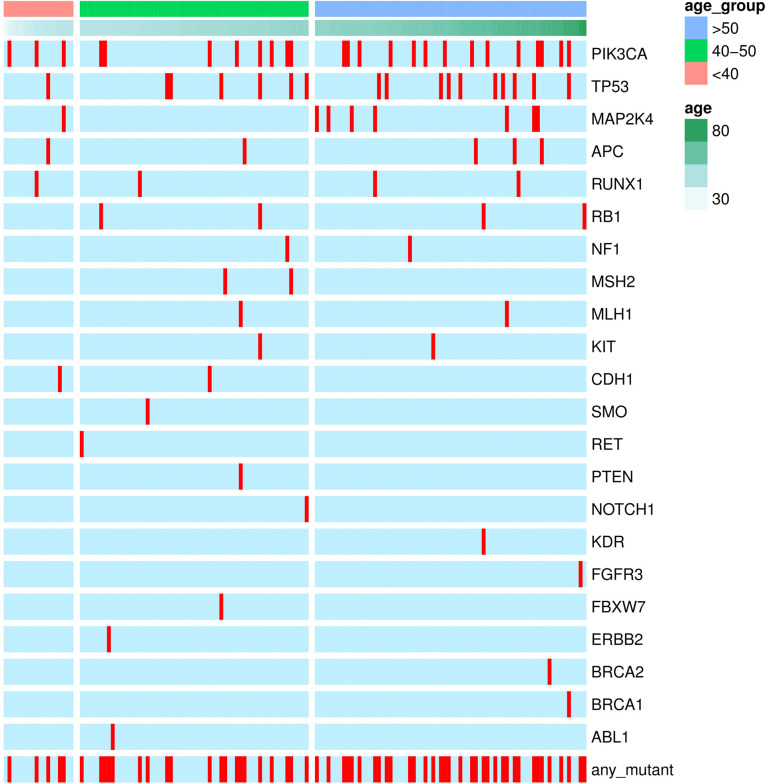
Genetic mutations identified by the RainDance™ gene panel in 147 breast cancers. The Oncoprint illustrated the distribution of somatic mutations according to age at diagnosis.

**Figure 4:**
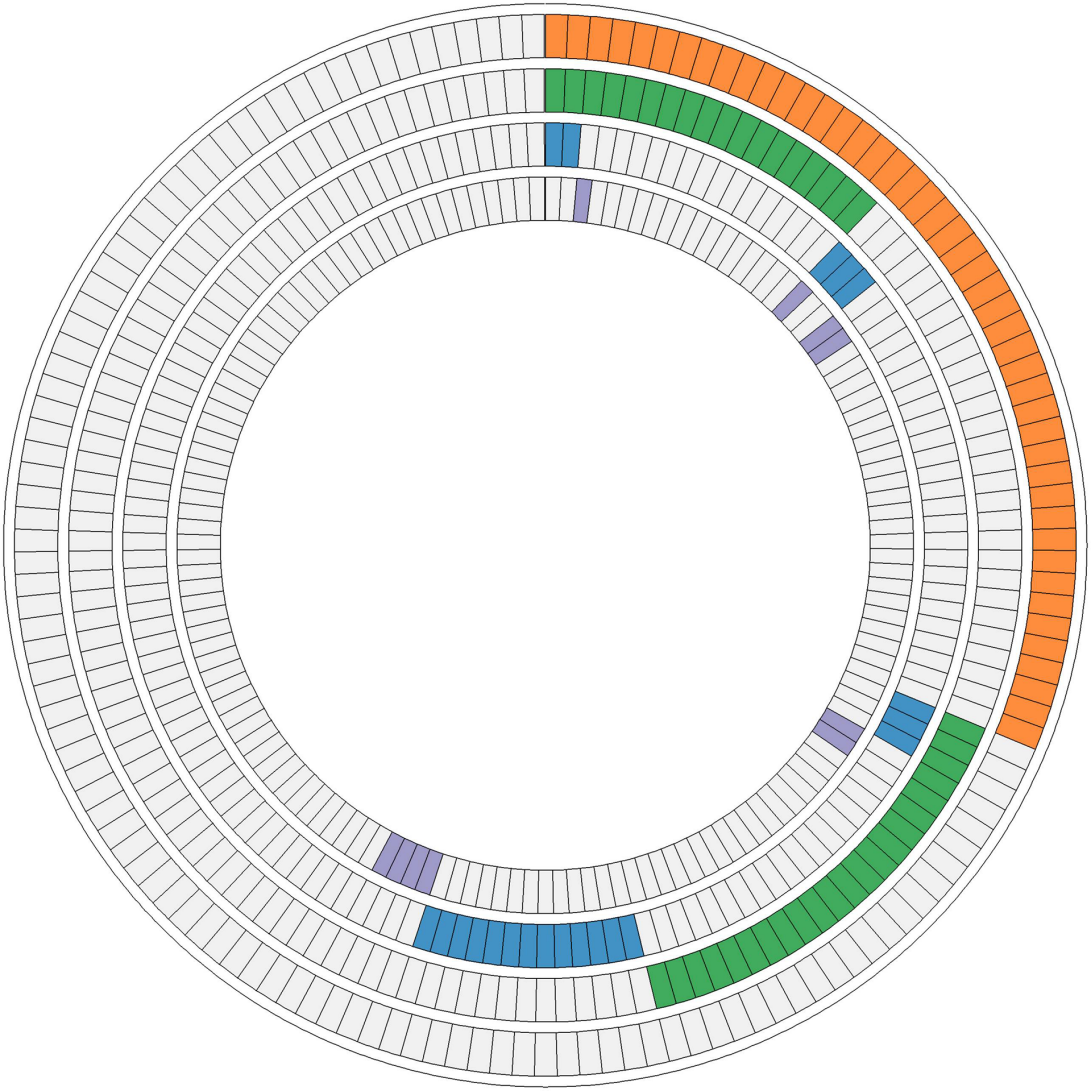
Ten interaction networks constructed from canonical maps including the mutations found in 147 luminal breast tumors. In the concentric circle diagram, tumors are arranged as radial spokes and categorized by their mutation status in each network (concentric ring color). Orange, genome integrity, proteolysis, and apoptosis; green, MAPK signaling and PI3K signaling; blue, RTK signaling and Hippo signaling; purple, Notch signaling, Hedgehog signaling, and Wnt signaling.

Fifty-four missense somatic mutations were identified in 22 genes of 85 patients. The next-generation sequencing analysis provided a quantitative assessment of the mutation frequency in each sample. We found that the missense somatic mutations found in the tumor samples were heterozygous mutations present with high allelic frequencies, ranging from 17% to 82% of the alleles in tissue samples. **Figure 2** shows that the most commonly mutated genes were PI3CA (5 mutations in 25 patients) and TP53 (17 mutations in 17 patients). The mean number of missense somatic mutations per tumor patient was 0.58 (range 0-4). The missense somatic variants found in the PIK3CA, TP53, MAP2K4 and APC genes were recurrent, with a frequency higher than 3% in the 147 breast cancer patients (**Figure 2**). These recurrent somatic mutations were validated by Sanger sequencing (**Table S3**). Finally, we found 37 functional mutations in 37 patients, and 7 patients had 2 functional mutations. **Figure 3** showed that older breast cancer patients accumulated more mutation and MAP2K4 mutations occurred in patients aged 50 years and older. DNA mismatch repair-related gene mutations were found in MSH2 (2 mutations in 2 patients), MLH1 (2 mutations in 2 patients), and BRCA1 (1 mutation in 1 patient). The cancer hotspot genes were involved in 16 canonical pathways. The 22 genes with missense somatic mutations represented 10 canonical pathways (**Table S4**). PolyPhen database predicts the functional effect and estimates the score difference between variants. Based on the scores of PolyPhen, NOTCH1, ABL1, SMO, APC, KDR, FGFR3, MLH1, PIK3CA, MSH2, RUNX1, TP53, BRCA1, NF1 and CDH1 were the 14 most significantly mutated genes (PolyPhen2_HDIV score> 0.9, **Table S4**). In the present study, APC was mutated in 5 breast cancer specimens, including two frameshift mutations, namely, p.R414S, and p.V1125A with poor outcomes in our breast cancer cohort, all of which had not been reported previously and were deemed to be very strong evidence of pathogenicity in breast cancer. However, no significant association between other genes and cancer prognosis was found in our study. APC was a negative regulator of WNT signaling pathway. Mutations in APC causing loss of APC tumor-suppressive functions were the primary mechanism for hyperproliferation in colorectal cancer. APC is both a marker for the development of chemotherapy resistance and a potential therapeutic target (22). **Figure 4** shows that most breast cancer patients did not have missense somatic mutations and that the missense somatic mutations detected in 85 patients affected a number of pathways enriched in genome integrity, apoptosis, MAPK signaling and PI3K signaling pathways. The IPA pathway analysis indicated that the 54 missense mutations were enriched in the hereditary breast cancer signaling pathway (P= 3.74E-13, **Table S5**), which is characterized by an inherited susceptibility to breast cancer due to mutations in high-penetrance susceptibility genes, including BRCA1, BRCA2, TP53 and PTEN.

### Correlating Mutations With Survival

**Figure 5:**
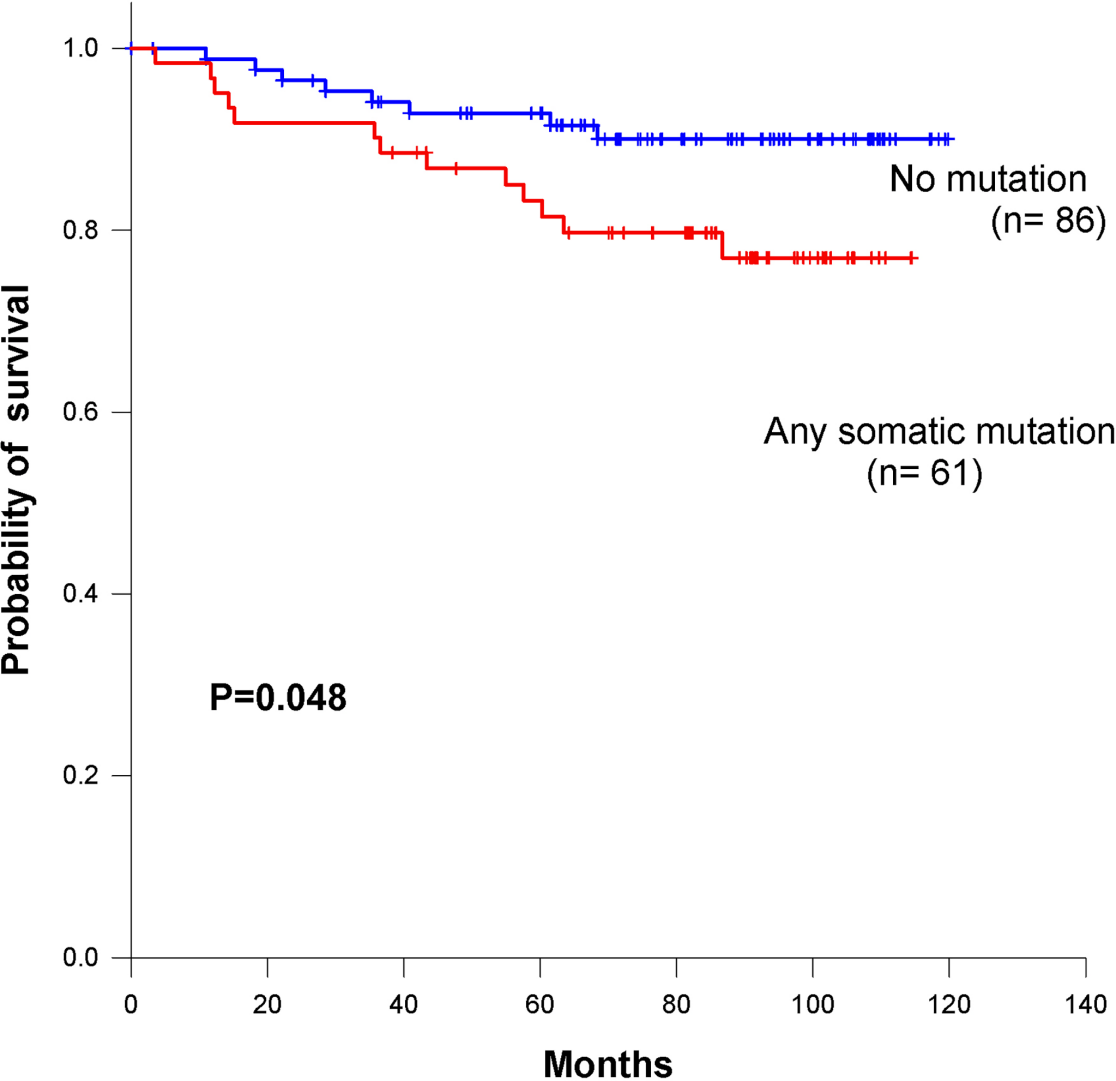
Kaplan–Meier survival curves for overall survival based on kinase signaling pathway in breast cancer patients.

We identified 101 somatic variants in breast cancer patients. In a Cox univariate analysis, only 54 nonsilent mutations were significantly associated with overall survival (P= 0.04, **Table 2**). All germline mutations were not significant in predicting overall survival in breast cancer patients (**Table S6**). Additionally, we identified that the 54 nonsilent somatic mutations were not significantly associated with overall survival prediction in breast cancer patients (log-rank P= 0.21, **Figure S1**). According to the functional pathways related to the identified genes, we distinguished two mutation signatures. One was a genomic instability signature including somatic mutations in RB1, TP53, BRCA1, BRCA2, MSH2, MLH1 and ABL1, and the other was a kinase signaling signature including somatic mutations in RET, PTEN, CDH1, MAP2K4, NF1, ERBB2, RUNX1, PIK3CA, FGFR3, KIT, KDR, APC, SMO, NOTCH1 and FBXW7. The kinase signaling signature significantly predicted overall survival in the 147 breast cancer patients (log-rank P=0.048 **Figure 5**), but the genomic instability signature did not significantly predict patient survival (log-rank P=0.51 **Figure S2**). Mutations in the kinase signaling pathway may have a negative prognostic role in survival in breast cancer. We compared the mutation frequency with TCGA database (**Figure S3**). We have provided the results in **Figure S4**. Kaplan-Meier survival analysis showed that there were significant in APC, FBXW7, FGFR3, KIT and RB1 groups. Despite there are some significant in those genes, only one or two patients have mutation in these genes except APC and RB1 gene.

**Table 2:** Ability of the somatic mutations to predict overall survival in breast cancer patients.

Variant type	Variant number	Hazard ratio	95% CI	P value	P value*
All	101	1.19	0.51 – 2.80	0.689	0.689
Non-silent	54	2.66	1.03 – 6.85	**0.043**	0.21
Silent	24	0.91	0.39 – 2.17	0.838	0.838
Non-coding	23	0.49	0.17 – 1.47	0.204	0.195

*: Log-rank P-Values.

### Correlating Mutations with Triple-Negative Breast Cancer

**Figure 6:**
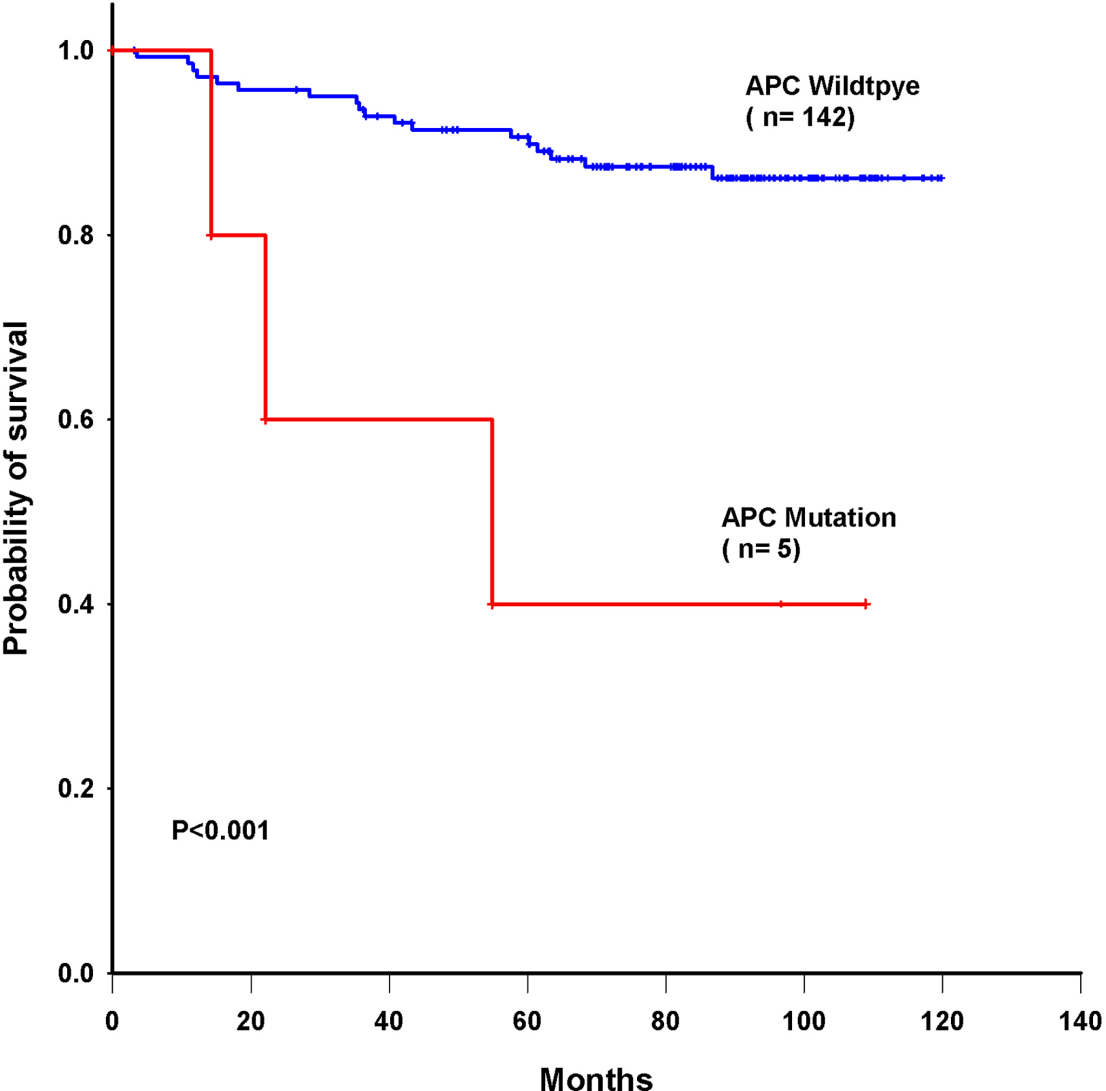
Kaplan–Meier survival curves for overall survival based on APC mutations in breast cancer patients.

Triple-negative breast cancer (TNBC) is defined as a breast tumor that is negative for estrogen receptor (ER), progesterone receptor (PR), and human epidermal growth factor receptor-2 (HER2). These tumors are usually marked by higher rates of metastases and recurrence and are insensitive to traditional hormone treatment and chemotherapy (23). We evaluated the relationship between the 22 genes with mutations and triple-negative breast cancer patients. **Table 3** indicates that mutations in the APC (P =0.006) and TP53 (P =0.045) genes were enriched in triple-negative breast cancer patients. Other genes were not enriched in any hormone receptor positive (ER/PR+) and HER2+ cohorts (**Table S7**). TP53-mutated breast cancer patients had significantly higher activity than TP53-wild-type patients. Meanwhile, we identified APC genes enriched with ER-negative and TNBC subtypes with statistical significance. In this subset analysis, mutations in novel codons that directly led to amino acid changes were related to the poorest overall survival. We further conducted multivariate Cox proportional hazard regression analysis with a signature containing the 37 novel mutations and other prognostic factors as predictors. We also evaluated the relationship between single gene mutation and survival in triple-negative breast cancer patients. A univariate Cox proportional hazard model showed that APC had the most significant impact on overall survival (P = 0.003) and significantly predicted overall survival in the 147 breast cancer patients (log-rank P<0.001, **Figure 6**).

**Table 3:** Evaluating associations between gene mutation status and triple-negative breast cancer patients by Fisher’s exact test.

Gene	Triple negative (N=29)	Non-TN (N=118)	P value
APC			**0.0055**
wild-type	25	117	
mutation	4	1	
TP53			**0.0451**
wild-type	22	108	
mutation	7	10	

## Discussion

An average of 1.02 to 1.66 somatic mutations per Mb in coding regions have been found in breast cancers (2, 12, 24), which translates into a mean of 56.9 (range 5-374) somatic mutations per tumor (25). Furthermore, significantly mutation genes in breast cancers have been identified, and these genes significantly affect pathways of functional or cellular processes, including the TP53 or PI3K pathway (e.g., PIK3CA, PTEN, and AKT1), MAPK/JNK signaling (e.g., MAP3K1, MAP2K4, and NF1), transcription factors and regulators (e.g., GATA3, RUNX1, and CBFB), splicing factors (e.g., SF3B1), and chromatin remodelers (e.g., MLL3 and ARID1A). It has been reported that only TP53, PIK3CA and GATA33 are consistently mutated in more than 10% of unselected breast cancers, while the remaining genes are mutated in less than 7% of patients, with a very long list of genes mutated in less than 1% of patients (2). Therefore, we applied targeted next-generation sequencing panel (with a high-depth sequencing method) for the detection of novel functional point mutations. The whole-genome sequences of the breast tumors indicated that the 10 most frequently mutated genes were TP53, PIK3CA, MYC, CCND1, PTEN, ERBB2, the ZNF703/FGFR1 locus on chromosome 8, GATA3, RB1 and MAP3K1[11]. However, we found that the 10 most frequently mutated genes were PIK3CA (17%), TP53 (12%), MAP2K4 (5%), APC (3.4%), RUNX1 (2.7%), RB1 (2.7%), NF1 (1.4%), MSH2 (1.4%), MLH1 (1.4%), KIT (1.4%), and CDH1 (1.4%). Consistent with previous mutational studies, PIK3CA and TP53 in our study were the most frequently mutated genes (12, 13, 26, 27). In our Asian breast cancer patient data, MAP2K4 had a higher mutation frequency than that seen in Caucasian breast cancer patients. Recently, the Taiwanese breast cancer study showed the high mutation frequency in PIK3CA, and TP53 (28, 29), but they did not investigate the mutations associated with patient survivals.

We indicated that missense somatic mutations were the most associated with survival. Germline mutations have been shown to impact tumor evolution and inheritance mechanisms (30, 31). Somatic mutation data in the Catalogue of Somatic Mutations in Cancer (COSMIC) (32) and TCGA provide somatic mutational landscapes across all cancer types. Missense somatic mutations in protein-coding regions can generate neoantigens (33, 34), which, in turn, could be associated with patient survival (35). Higher somatic mutation and neoantigen burdens are correlated with poor progression-free survival in multiple myeloma (36). This is consistent with our findings.

Mutations of the APC tumor-suppressor gene are thought to regulate beta-catenin within the Wingless/Wnt signal transduction pathway (37). We found that the number of missense somatic mutations and mutations in APC (V1125A and R414S) were independent predictors for the overall survival in our breast cancer patients. Here, we first reported the identification of a novel APC mutation in Taiwanese breast cancers patients. Furthermore, we also revealed that mutations in the TP53 and APC genes were enriched in TNBC. The TP53 mutation frequency was higher in TNBC than in other types of breast cancer (38). TP53-mutated breast cancer patients had significantly higher activity than TP53-wild-type patients. Furthermore, we explored the molecular markers correlated with the differences in immune activities between TP53-mutated and TP53-wild-type patients (39). TP53 mutation regulates immune responses. This pathway is involved in host immunity, influencing both innate and adaptive immune responses (40). Wnt signaling dysfunction has been reported to mediate the progression of triple-negative breast cancer (41). A proteomics study showed that TNBC tumors with APC mutations expressed median levels of β-catenin that were 4-fold higher than those in tumors without APC mutations (42). β-Catenin was required for the tumor cell motility in triple-negative breast cancer cells (43). Wnt/β-catenin pathway activation has been implicated in stem cell self-renewal, maintenance, and differentiation (44, 45). We first reported that APC somatic mutations (V1125A and R414S) were enriched in TNBC and associated with reduced overall survival in all breast cancer patients. These mutations may identify new biomarkers for breast cancer patients in the future.

## Conclusions

In conclusion, we have analyzed targeted sequencing data generated from 147 Taiwanese breast cancer patients by using RainDance Cancer Hotspot Panel. We observed 54 somatic functional point mutations distributed across 22 genes in 85 patients. Mutations of APC and TP53 were enriched in TPNBC and patients with Patients with APC mutations had significantly poorer overall survival than patients without APC mutations. However, the mutations in kinase genes had significantly poorer overall survival than patients without kinase gene mutations. The results indicated that APC variant V1125A and R414S might be the oncogenic and the kinase genes association of survival in breast cancer patients which provides novel target for the development of breast cancer and identifies APC as a novel putative target for targeted therapy.

## Data Availability Statement

The datasets presented in this study can be found in online repositories. The names of the repository/repositories and accession number(s) can be found in the article/**Supplementary Material**.

## Ethics Statement

Written informed consent was not obtained from the individual(s) for the publication of any potentially identifiable images or data included in this article.

## Author Contributions

P-SY, C-LL, K-CL and Y-CH made substantial contributions to conception and design. C-LL, P-SY, and Y-CC were responsible for clinical management and interpreted clinical data. P-SY, K-CL, Y-TC, and Y-CH wrote the manuscript. YCH, P-SY and C-FL interpreted the data and analyzed the data. All authors gave final approval for the manuscript.

## Conflict of Interest

The authors declare that the research was conducted in the absence of any commercial or financial relationships that could be construed as a potential conflict of interest.

## Publisher’s Note

All claims expressed in this article are solely those of the authors and do not necessarily represent those of their affiliated organizations, or those of the publisher, the editors and the reviewers. Any product that may be evaluated in this article, or claim that may be made by its manufacturer, is not guaranteed or endorsed by the publisher.
