# Supplementary material for: Association of Pathway Mutations With Survival in Taiwanese Breast Cancers
Source: Front Oncol. 2022 Jul 22;12:819555. doi: 10.3389/fonc.2022.819555 (PMC9354680; doi:10.3389/fonc.2022.819555)

Figure S1 Kaplan–Meier survival curves for overall survival analysis based on of somatic missense mutation numbers in breast cancer patients.

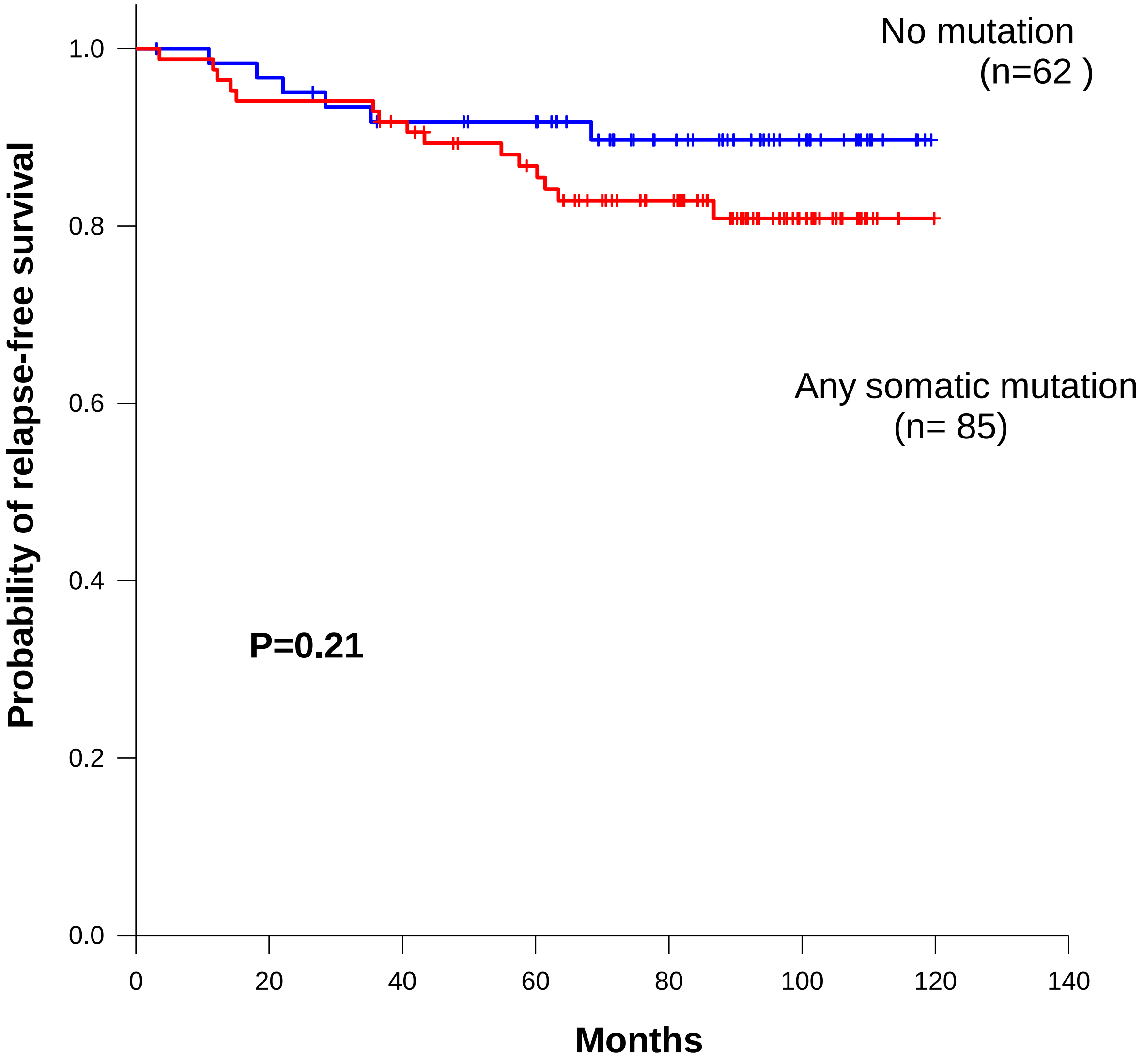

Supplement: Supplementary file 3 [file DataSheet_3.zip › Supplementary Figure 1.PDF]
