# Supplementary material for: Association of Pathway Mutations With Survival in Taiwanese Breast Cancers
Source: Front Oncol. 2022 Jul 22;12:819555. doi: 10.3389/fonc.2022.819555 (PMC9354680; doi:10.3389/fonc.2022.819555)
Supplement: Supplementary file 3 [file DataSheet_3.zip › Supplementary Figure 4.PDF]

Figure S4 Kaplan–Meier survival curves for overall survival analysis based on of gene mutation numbers in breast cancer patients.

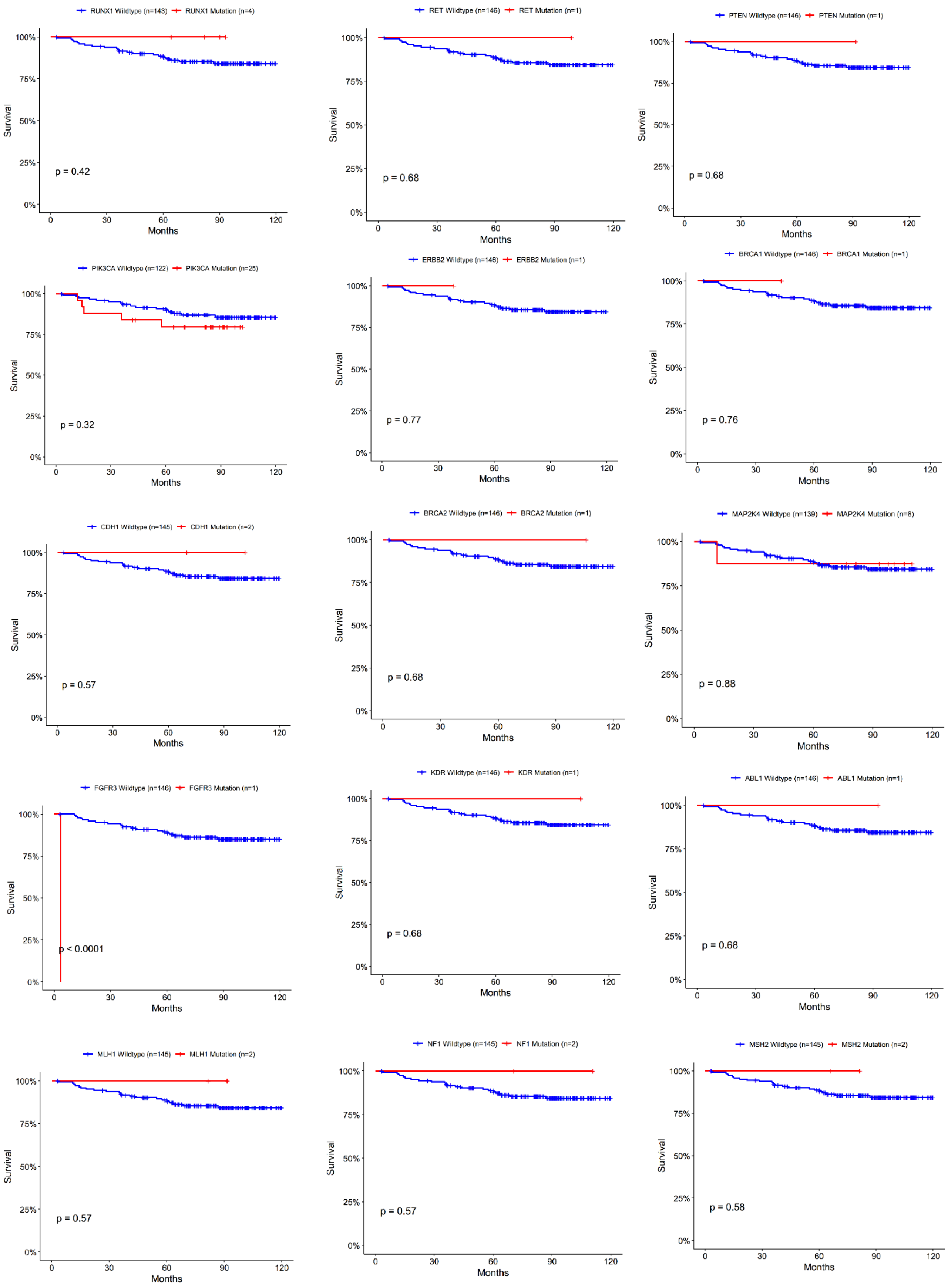

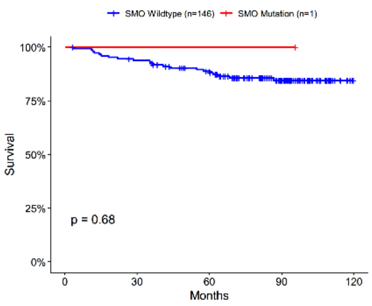
SMO Wildtype (n=146)
SMO Mutation (n=1)
Survival
100%
75%
50%
25%
0%
p = 0.68
0
30
60
90
120
Months

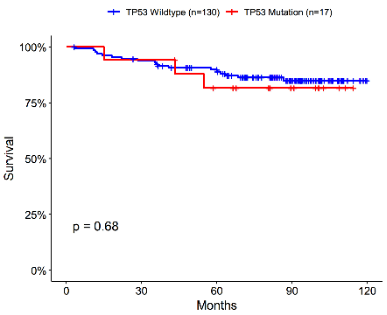
TP53 Wildtype (n=130)
TP53 Mutation (n=17)
Survival
100%
75%
50%
25%
0%
p = 0.68
0
30
60
90
120
Months

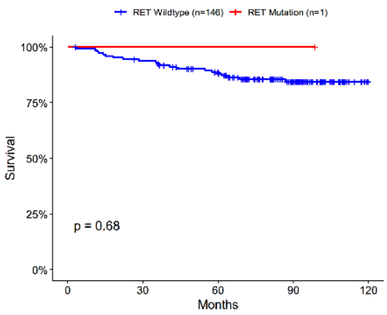
RET Wildtype (n=146)
RET Mutation (n=1)
Survival
100%
75%
50%
25%
0%
p = 0.68
0
30
60
90
120
Months

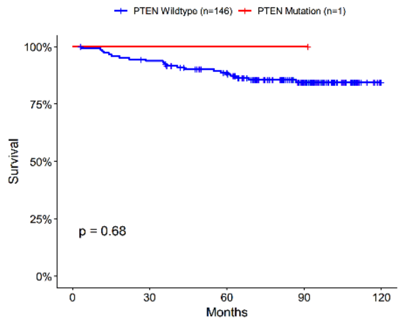
PTEN Wildtype (n=146)
PTEN Mutation (n=1)
Survival
100%
75%
50%
25%
0%
p = 0.68
0
30
60
90
120
Months

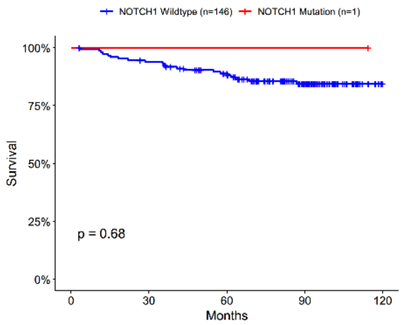
NOTCH1 Wildtype (n=146)
NOTCH1 Mutation (n=1)
Survival
100%
75%
50%
25%
0%
p = 0.68
0
30
60
90
120
Months

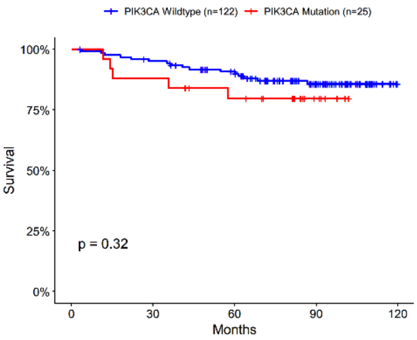
PIK3CA Wildtype (n=122)
PIK3CA Mutation (n=25)
Survival
100%
75%
50%
25%
0%
p = 0.32
0
30
60
90
120
Months

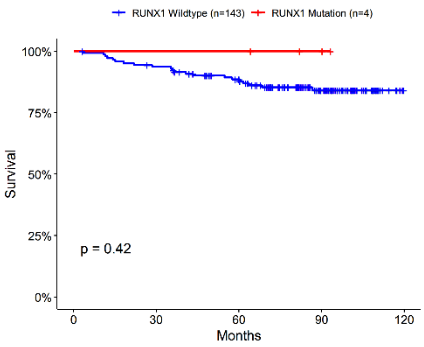
RUNX1 Wildtype (n=143)
RUNX1 Mutation (n=4)
Survival
100%
75%
50%
25%
0%
p = 0.42
0
30
60
90
120
Months
